# Low-light activity in fallow deer shows sex-specific seasonal changes in a managed landscape

**DOI:** 10.1007/s13364-026-00903-8

**Published:** 2026-09-29

**Authors:** Akke de Lange, Luc H. W. T. Geelen, W. Daniel Kissling

**Affiliations:** 1 Institute for Biodiversity and Ecosystem Dynamics (IBED), University of Amsterdam, P.O. Box 94240, Amsterdam, 1090 GE The Netherlands; 2 Waternet, Vogelenzangseweg 21, Vogelenzang, 2114 BA the Netherlands

**Keywords:** Diel activity, Nocturnality, Sexual segregation, Behavioural ecology, Fallow deer (*Dama dama*), Camera trapping

## Abstract

Diel activity patterns of large herbivores vary with environmental conditions, human disturbance, and wildlife management, yet population-level analyses may obscure sex- and season-specific patterns. We examined seasonal variation and sexual segregation in diel activity of fallow deer (*Dama dama*) in a managed coastal dune system in the Netherlands characterised by substantial but temporally variable daytime recreation, absence of large predators, and female-focused culling. Using a camera-trap network (49 sites; 1,490 camera-trap days), we analysed 4,114 sexed detections collected during three seasons (rut, post-rut, and birthing). Diel activity was assessed using a diel niche framework accounting for seasonal variation in light availability, complemented by equinoctially anchored kernel density estimates and activity overlap metrics. At the population level, fallow deer consistently selected twilight and night, particularly during rut and post-rut. Sex-specific analyses revealed divergence outside the rut. Females exhibited persistent nocturnality, especially post-rut, whereas males increased twilight and daytime activity during the birthing season, showing that the spring increase in daytime activity was largely male-driven. Activity overlap between sexes was highest during rut and declined thereafter, indicating increasing sexual segregation in diel timing. These results demonstrate that pooling sexes masks sex- and season-specific activity patterns. Although these patterns occurred in a landscape characterised by daytime recreation and sex-biased management, their underlying mechanisms cannot be disentangled from intrinsic behavioural and energetic constraints. Our findings highlight the importance of incorporating sex- and season-specific activity patterns into wildlife monitoring and management.

**Supplementary Information:**

The online version contains supplementary material available at https://doi.org/10.1007/s13364-026-00903-8.

## Introduction

Diel activity patterns are a fundamental component of animal ecology, shaping energy balance, thermoregulation, foraging behaviour, social interactions, and reproduction (Kronfeld-Schor and Dayan 2003; Cox et al. 2021; Devarajan et al. 2025). Animals must continuously balance the benefits of activity against risks that vary predictably over the 24-hour cycle, including predation, weather, and human disturbance (Brivio et al. 2016; Chitwood et al. 2022; Delisle et al. 2024). In human-dominated landscapes, this balance is increasingly modified by recreation, hunting, and wildlife management, which can function as temporally structured sources of risk (Gaynor et al. 2018). Consequently, diel activity is often highly plastic rather than a fixed species trait, emerging from local combinations of environmental conditions, disturbance regimes, and management practices (Coppes et al. 2017; Ikeda et al. 2019; Millien et al. 2024; Torretta et al. 2025). Understanding when animals are active is therefore not only central to behavioural and population ecology, but also has direct implications for wildlife monitoring, impact assessment, and management. Shifts in activity timing can decouple the perceived presence of animals from their actual ecological impacts, for example when monitoring relies on daytime observations while animals increasingly forage nocturnally in specific habitats (Coppes et al. 2017; Chitwood et al. 2022). Understanding how mammals adjust activity timing under anthropogenic pressure is therefore central to behavioural ecology as well as applied wildlife management.

Large herbivores, and ungulates in particular, show pronounced flexibility in diel activity in response to human disturbance and management. Across species and habitats, recreation, hunting, and culling have repeatedly been shown to shift ungulate activity toward twilight and night-time periods, effectively reducing temporal overlap with humans (Coppes et al. 2017; Chitwood et al. 2022; Millien et al. 2024; Torretta et al. 2025). Such shifts are often interpreted within a “landscape of fear” framework, whereby animals adjust their behaviour in response to spatially and temporally varying perceptions of risk arising from predators, humans, and associated risk cues (Ciuti and Apollonio 2008; Gaynor et al. 2019; Suraci et al. 2019). In human-dominated landscapes, wildlife responses to humans can range from tolerance to avoidance depending on the type of human activity and previous exposure to lethal and non-lethal interactions (Zenth et al. 2026). Where humans are perceived as a source of risk, ungulates may reduce temporal overlap with human activity by concentrating activity during twilight and night-time periods, with responses potentially strengthened by hunting pressure (Coppes et al. 2017; Ikeda et al. 2019; Torretta et al. 2025; Boer-Cueva et al. 2026). These behavioural responses are ecologically consequential. Ungulates may respond to human disturbance through a combination of temporal avoidance (shifting activity into low-disturbance periods) and spatial avoidance (using areas with lower human activity), with the relative importance of these strategies varying among systems and seasons (Coppes et al. 2017; Chitwood et al. 2022; Millien et al. 2024). Such spatio-temporal adjustments can alter patterns of foraging, habitat use, and herbivory pressure, with cascading effects on vegetation and biodiversity (Jia et al. 2018). Understanding how ungulates adjust their diel activity in response to human disturbance and management is therefore critical for interpreting population-level patterns and for evaluating the effectiveness and unintended consequences of wildlife management in human-modified landscapes.

Diel activity in ungulates is also not static across the year, but varies seasonally in response to changes in photoperiod, weather, forage availability, and reproductive demands (Brivio et al. 2016; Delisle et al. 2024). Camera-trap and telemetry studies have documented substantial seasonal shifts in activity patterns across temperate landscapes, often expressed as changes in the timing and intensity of activity peaks rather than complete switches between diel phenotypes (e.g. Caravaggi et al. 2018). In fallow deer, recent work indicates increased diurnality during the spring birthing period, while activity remains more crepuscular or nocturnal during rut and post-rut, although the magnitude and direction of these shifts differ among populations and study areas (Caravaggi et al. 2018; Zaman et al. 2022; Barton 2023). Such seasonal patterns are shaped by local combinations of climatic conditions, human disturbance, and management regimes, rather than reflecting fixed species-level traits (Apollonio and Chirichella 2023). Despite this documented seasonal plasticity, many studies summarise activity at the population level by pooling detections across seasons, potentially obscuring biologically meaningful temporal structure. Pooling data can mask shifts in activity timing that are critical for understanding when and how animals interact with their environment, particularly in systems where disturbance and management pressures vary seasonally. Resolving seasonal variation in diel activity is therefore essential for accurately interpreting ungulate behaviour and for considering observed activity patterns in relation to potential ecological and management drivers.

In addition to seasonal plasticity, ungulates commonly exhibit pronounced sexual segregation in space, habitat use, and behaviour outside the mating period, reflecting differences in body size, energetic requirements, reproductive roles, and risk sensitivity (Bowyer and Kie 2004; Ciuti et al. 2004, 2006; Bowyer 2022). In fallow deer, males and females aggregate briefly during the rut but segregate for much of the remainder of the year, with females bearing the energetic costs of gestation and lactation and males contributing no parental care (Ciuti et al. 2006; Imperio et al. 2020; Barton 2023). These differences are expected to translate into sex-specific diel activity patterns, particularly under conditions of disturbance or perceived risk, as females in sexually dimorphic ungulates often show stronger avoidance behaviour and greater sensitivity to risk than males (Ciuti et al. 2004; Ciuti and Apollonio 2008; Gallotta et al. 2025). Despite strong theoretical and empirical support for sexual segregation, many studies of ungulate diel activity pool detections across sexes, implicitly assuming a shared temporal niche and potentially obscuring both sex-specific activity patterns and differences in behavioral variability among individuals. This approach risks masking sex-specific activity patterns, especially in landscapes where management practices impose unequal risk on males and females. Sex-biased hunting or culling, in particular, can create asymmetric risk regimes that may contribute to sex-specific behavioural responses (Ciuti and Apollonio 2008; Ikeda et al. 2019; Chitwood et al. 2022). Resolving diel activity by sex is therefore essential for understanding how ungulates respond to human disturbance and management in managed landscapes.

The Amsterdamse Waterleidingduinen (AWD) in the Netherlands provides an informative setting for examining diel activity in a human-dominated and managed landscape. This area is characterised by substantial but temporally variable recreational use, long-standing female-focused population control, and the absence of large natural predators (Kissling et al. 2024; van der Spek 2024). Recreational access is restricted to daylight hours, whereas its intensity varies temporally. Population control provides an additional, seasonal and sex-biased source of human-mediated risk. Although the effects of recreation, culling and intrinsic seasonal and sex-specific processes cannot be disentangled in this observational study, the AWD provides a valuable opportunity to examine seasonal and sex-specific diel activity patterns within this management context.

In this study, we quantified seasonal and sex-specific variation in the diel activity of European fallow deer in a highly managed, human-dominated landscape. Using an established grid-based camera-trap network in the Amsterdamse Waterleidingduinen (Kissling et al. 2024), we analysed fallow deer activity during three biologically defined seasons—rut, post-rut, and birthing—and explicitly separated analyses by sex. We first classified diel activity using a model-based diel niche framework that accounts for seasonal variation in light availability, focusing on selection-based diel niche classification at both the population and seasonal levels (Gerber et al. 2024). We then examined seasonal variation in continuous activity patterns using equinoctially anchored kernel density estimates, before assessing sex-specific activity patterns and temporal overlap within each season. Analyses were first conducted at the population level, reflecting the way activity patterns are commonly summarized in monitoring and management contexts, before explicitly evaluating sex-specific patterns. Based on previous studies showing increased nocturnality of ungulates in response to human disturbance and management, seasonal shifts in fallow deer activity, and stronger risk sensitivity in females than males (Ciuti et al. 2004; Ciuti and Apollonio 2008; Caravaggi et al. 2018; Ikeda et al. 2019; Barton 2023; Gallotta et al. 2025), we predicted that (1) fallow deer would exhibit an overall bias toward twilight and night activity, (2) seasonal shifts in diel activity would occur outside the rut, and (3) females would show stronger and more persistent nocturnality than males, resulting in increased sexual segregation in activity timing, particularly during the birthing period when females invest into parental care. By resolving diel activity across biologically meaningful seasons and sexes, this study aims to clarify seasonal and sex-specific variation in low-light activity and to highlight the importance of sex-specific analyses for wildlife monitoring and management.

## Methods

### Study area

The study was conducted in the Amsterdamse Waterleidingduinen (AWD; 52°20’10.3"N, 4°31’49.9"E), a 34 km² coastal dune reserve along the western coast of the Netherlands and part of the Natura 2000 site Kennemerland-Zuid (NL1000012). The area comprises a mosaic of dune grasslands, scrublands, forests, and open sand habitats (Kissling et al. 2024). The AWD is also a major recreational area, receiving approximately one million visitors annually and primarily serving local and regional recreation. Recreational use varies seasonally. Waternet ticket-sales records for the three sampling months show that 16,475 day-admission tickets were sold in April 2024, 30,075 in October 2024, and 13,198 in January 2025 (Waternet, unpublished data), corresponding to the birthing, rut, and post-rut sampling periods, respectively. These figures provide an index of seasonal visitation rather than complete visitor counts because not all visitors purchase a day-admission ticket. Visitor intensity also varies among weekdays and weather conditions. Public access is restricted to the period between sunrise and sunset; thus, while the intensity of recreational use varies, its diel timing is largely restricted to daylight hours. Large natural predators are absent, and the area is almost entirely fenced, limiting immigration and emigration of large herbivores.

European fallow deer (*Dama dama*), introduced in the late twentieth century, are abundant and exert substantial grazing, browsing, and trampling pressure, contributing to vegetation change and biodiversity loss (van der Spek 2024). To reduce these impacts, a population control programme was initiated in 2016 following estimated spring densities of 190–220 deer km⁻² (van der Spek 2024). Population control consists of annual culling between 1 November and 1 March, typically on four days per week, conducted by three teams of reserve wardens using daytime shooting and focused primarily on females and fawns to reduce population growth (van der Spek 2024). Since its implementation, the programme has removed between 547 and 2,315 deer annually and has contributed to reductions in population size and shifts in population structure (van der Spek 2024).

Recreational use therefore represents a recurrent source of daytime disturbance whose intensity varies temporally, whereas population control imposes a seasonal and sex-biased source of lethal risk. Together with the absence of large predators, these factors characterise the human-dominated context in which fallow deer activity was studied.

### Focal species

The European fallow deer (*Dama dama*) is a sexually dimorphic ungulate that exhibits substantial behavioural plasticity in space use and diel activity (Ciuti et al. 2004, 2006; Ciuti and Apollonio 2008; Imperio et al. 2020; Barton 2023). Outside the rut, fallow deer show pronounced sexual segregation linked to differences in body size, energetic demands, reproductive roles, and risk sensitivity. Females bear the costs of gestation and lactation and often exhibit stronger avoidance behaviour and sensitivity to perceived risk, whereas males generally show greater risk tolerance (Ciuti et al. 2004; Ciuti and Apollonio 2008; Gallotta et al. 2025). In the AWD, long-standing culling focused on females and fawns creates sex-biased exposure to lethal risk, making fallow deer an appropriate focal species for examining seasonal and sex-specific diel activity responses to human disturbance and wildlife management.

### Data collection and transmission

**Fig. 1:**
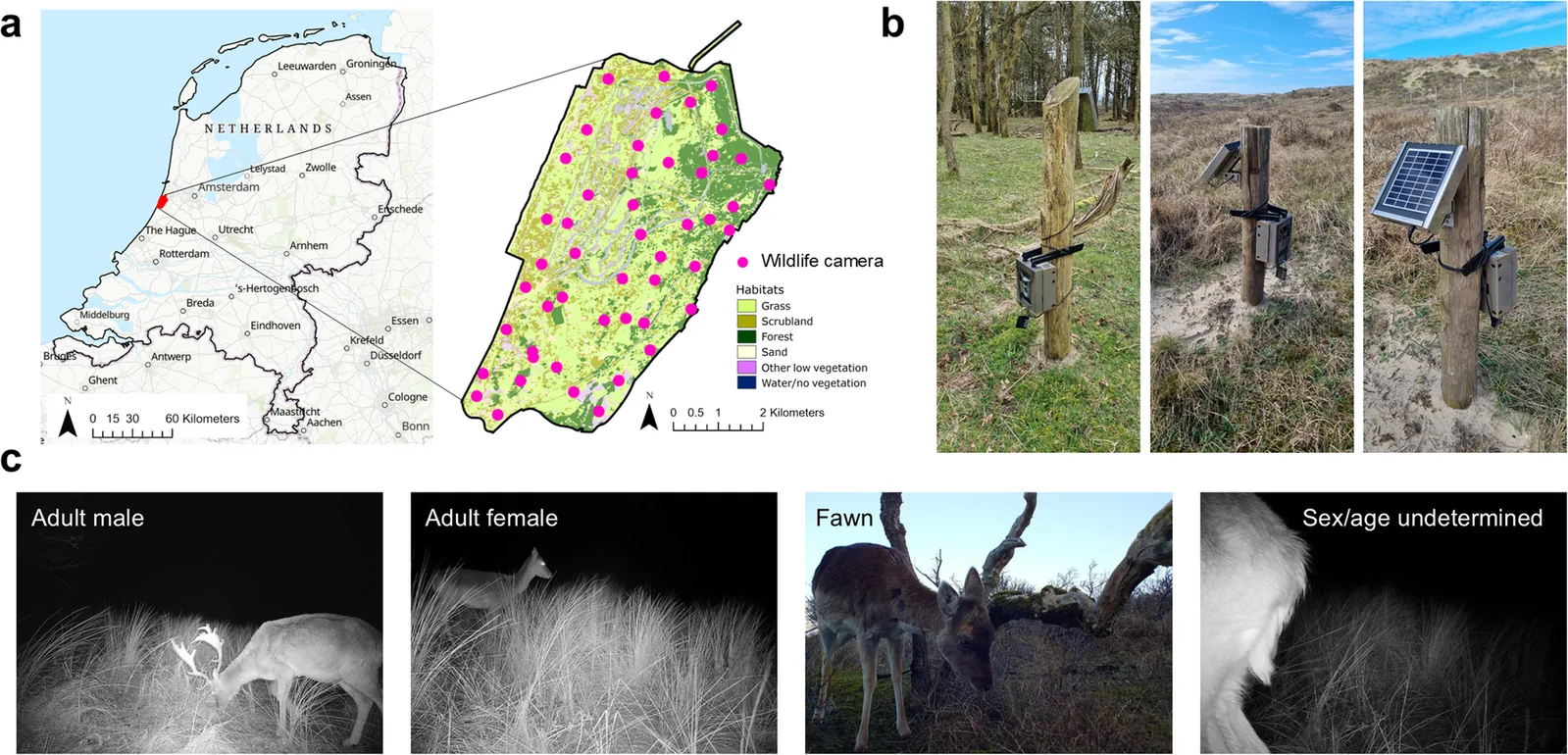
Study area, camera traps, and focal species. (**a**) Location of the Amsterdamse Waterleidingduinen within the Netherlands and distribution of the wildlife camera-trap network (*n* = 49 cameras). (**b**) Examples of camera-trap deployment, showing solar-powered cameras mounted with security cages on wooden poles at 30–50 cm height (photo credits: W. Daniel Kissling). (**c**) Examples of images showing sex and age classes of European fallow deer. The illustrated photos are from pilots in the study area (Evans et al. 2024) and publicly available with a CC BY 4.0 license from the Global Biodiversity Information Facility (https://doi.org/10.15468/u88x5y)

Data were collected using an automated camera-trap network established in the AWD in 2023 (Fig. 1; Kissling et al. 2024), developed in the context of the project ARISE (Authoritative and Rapid Identification System for Essential biodiversity information; van Ommen Kloeke et al. 2025). The network consists of 65 wireless cameras (Wilsus Tradenda 4G Wireless and Snyper Commander 4G Wireless; Wildlife Monitoring Solutions, the Netherlands), equipped with passive infrared (PIR) sensors and infrared flash for nocturnal imaging. Cameras were configured to record five images per trigger event and automatically transmit images via the cellular network. Further technical details of the camera-trap network are provided in Kissling et al. (2024).

The overall sampling design combined a grid-based sampling network, monitoring along a long-term rabbit survey route, and paired sampling inside and outside fallow deer exclosures (Kissling et al. 2024). For the present study, we used only cameras located outside the fallow deer exclosures (*n* = 49; Fig. 1a), because these areas were accessible to deer and therefore suitable for analyzing deer activity patterns. Cameras located inside exclosures (*n* = 16), where deer access was prevented, were not included.

Cameras were deployed in a grid-based design (at least one per 1 × 1 km cell), supplemented by placements along the rabbit monitoring route, ensuring near-proportional coverage of major habitat types. Following established camera-trapping guidelines (Wearn and Glover-Kapfer 2017), cameras were mounted approximately 30–50 cm above ground, oriented northwards, deployed off-trail, and left unbaited (Kissling et al. 2024). Camera locations were fixed throughout the study.

To capture seasonal variation, we selected images from three biologically defined seasons based on the fallow deer reproductive cycle: rut (1–10 October 2024), post-rut (1–10 January 2025), and birthing (1–10 April 2024) (Ciuti et al. 2006; Barton 2023). Cameras operated continuously, and sampling the same 10-day period in each season ensured comparable sampling effort, resulting in a total effort of 1,490 camera-trap days (49 cameras × 10 days × 3 seasons).

### Image annotation and sex classification

**Fig. 2:**
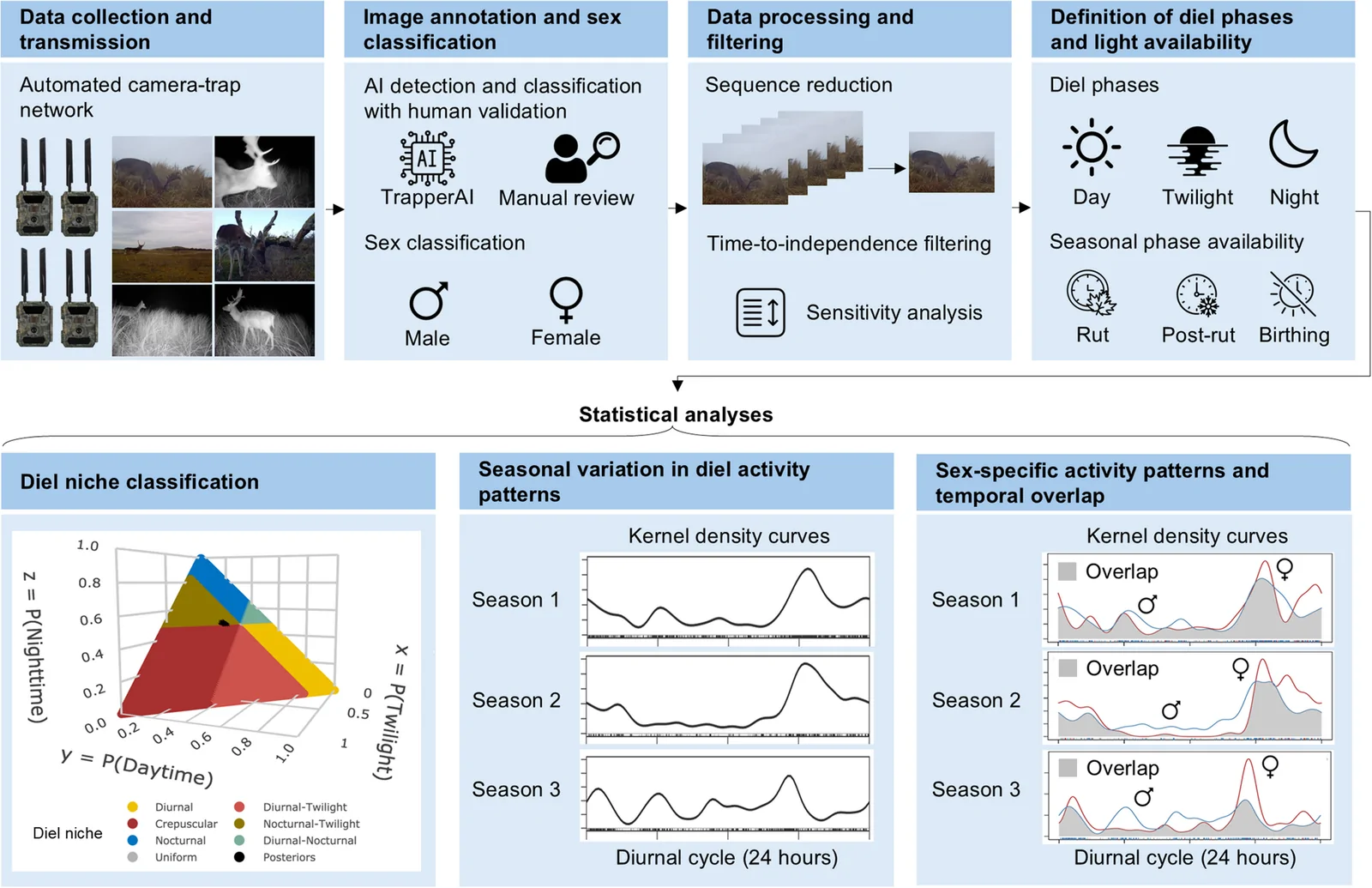
Workflow from camera-trap data collection and pre-processing steps to statistical analyses of fallow deer diel activity. The workflow illustrates successive steps including data collection and transmission, image annotation and sex classification, data processing and filtering, definition of diel phases and light availability and statistical analyses, comprising diel niche classification, seasonal variation in diel activity patterns, and sex-specific activity patterns and temporal overlap. Black icons from Flaticon (https://www.flaticon.com/)

After data collection and transmission (Fig. 2), images were annotated using a custom-built sensor portal for the AWD camera network (Kissling et al. 2024). Image annotation was supported by the TrapperAI Species Classifier (TrapperAI v02.2024), a deep-learning model developed by the Open Science Conservation Fund (OSCF) for automated classification of European mammal species. All automated classifications (including fallow deer and other species) were manually reviewed, and only images with confident fallow deer detections were retained. The classifier was used to accelerate the annotation workflow, whereas all records included in the analyses were verified by human observers.

The sex (male, female and undetermined) of each fallow deer image was classified (see examples in Fig. 1c). Sex and age class were assigned based on visible morphological characteristics, including body size and shape, antler presence or development, and secondary sexual traits (Langelaar 2024; de Lange 2025). Sex was recorded only when confidently identifiable; ambiguous cases were reviewed by a second observer and excluded from sex-specific analyses if uncertainty remained. Images were also annotated for behavioural state, and detections classified as resting or sleeping were excluded. Annotated metadata were exported for downstream analyses via the portal’s application programming interface (Kissling et al. 2024).

### Data processing and filtering

Annotated records were processed in R (version 4.5.0) to obtain independent activity detections (Fig. 2). Cameras recorded five-image sequences per trigger at 0.35 s intervals with a 30 s delay. Each sequence was reduced to a single detection (Fig. 2), reducing pseudo-replication while retaining activity timing information. In case multiple individuals were present, the frame with the highest number of fallow deer was selected.

 Detections lacking confident sex classification were excluded from sex-specific analyses. Remaining detections were assigned to sex and season based on timestamps, using a standard datetime format (i.e. POSIXct). This resulted in a final dataset of 4,114 detections (949 females, 3,165 males; Table S1).

 Because time-to-independence filtering can bias activity-pattern analyses (Peral et al. 2022), we performed a sensitivity analysis to evaluate its effect using a 15-minute threshold supported by the data (Text S1, Fig. S1). Filtering substantially reduced sample size and inferential precision (Tables S1–S3); therefore, all main analyses were conducted without filtering. Full sensitivity analyses are provided in Text S1.

### Definition of diel phases and light availability

Each detection was assigned to one of three diel phases: day, twilight, or night (Fig. 2). Sunrise and sunset times were calculated using the *suncalc* package (Thieurmel and Elmarhraoui 2022). Twilight was defined as two-hour windows centred on sunrise and sunset (Caravaggi et al. 2018); remaining daylight and dark hours were classified as day and night, respectively.

 Seasonal phase availability was calculated as the proportion of the 24-hour cycle occupied by each phase and used to account for photoperiod variation in activity classification (Nouvellet et al. 2012; Gerber et al. 2024). To visualise activity independently of clock time, timestamps were transformed to relative sun time using equinoctial anchoring (Nouvellet et al. 2012). Phase availability and photoperiod values are provided in Table S4.

### Diel niche classification

Diel activity was classified using the selection-hypothesis framework implemented in the *Diel.Niche* R package (Gerber et al. 2024). This framework compares observed diel phase use with phase availability, allowing discrimination between behavioural selection and photoperiod-driven shifts (Nouvellet et al. 2012; Gerber et al. 2024). Diel niches were classified separately by season and at the population level.

Because *Diel.Niche* does not support aggregation across seasons, population-level classification was derived using season-weighted averages of phase-specific detection probabilities, with weights proportional to each season’s contribution to total detections. These estimates, together with calendar-weighted phase availability values, were supplied to the *diel.fit* function to classify the population-level diel niche.

 Candidate hypotheses included uniform (i.e. equal use of diel phases), diurnal, nocturnal, crepuscular (= twilight), and mixed strategies (nocturnal– twilight, diurnal– twilight, and diurnal–nocturnal), defined by constraints on relative phase use. Hypotheses were compared using posterior probabilities from a Bayesian multinomial framework, and the most supported hypothesis was retained. Full hypothesis definitions, constraints, and prior specifications are provided in Text S2 and Table S5.

### Seasonal variation in diel activity patterns

Seasonal variation in diel activity was further examined using continuous activity pattern visualisation based on circular kernel density estimation of detection times. To ensure comparability across seasons with differing photoperiods, all timestamps were transformed to relative sun time using equinoctial anchoring, with sunrise fixed at π/2 and sunset at 3π/2 (Nouvellet et al. 2012). Kernel density estimates were calculated using the *overlap* package in R (Ridout and Linkie 2009; Meredith et al. 2024), which is specifically designed for analysing circular time-of-day data. Activity distributions were estimated separately for each biologically defined season (rut, post-rut, and birthing) at the population level (males and females combined).

These season-specific activity kernels were used to visualise shifts in the timing and shape of activity peaks across the year and to assess whether seasonal changes reflected redistribution of activity within low-light periods or shifts toward daytime activity. Kernel density plots were interpreted in conjunction with selection-based diel niche classifications to ensure consistency between continuous activity patterns and categorical diel phase use.

### Sex-specific activity patterns and temporal overlap

Sex-specific diel activity patterns were analysed by estimating separate kernel density distributions for males and females within each season, again using equinoctially anchored relative sun time and circular kernel density estimation implemented in the *overlap* package (Ridout and Linkie 2009; Meredith et al. 2024). These sex-specific kernels allowed visual comparison of activity timing and peak structure between males and females within the same seasonal context.

Temporal overlap between activity patterns was quantified using the coefficient of overlap (Δ̂), which measures the shared area under two kernel density curves and ranges from 0 (no overlap) to 1 (complete overlap) (Ridout and Linkie 2009). Overlap was estimated using the Δ̂4 estimator, which is recommended for sample sizes greater than 50 (Rowcliffe 2023; Meredith et al. 2024). Overlap analyses were conducted for comparisons between sexes within each season. Uncertainty around overlap estimates was quantified using non-parametric bootstrap resampling with 1,000 iterations, implemented in the *activity* package (Rowcliffe 2023), to derive 95% confidence intervals (Peral et al. 2022; Iannino et al. 2025).

Kernel density plots and overlap metrics were interpreted alongside diel niche classifications to provide a complementary continuous assessment of sex- and season-specific variation in activity timing.

### Software and reproducibility

All analyses were conducted in R (version 4.5.0; R Core Team 2025). Data processing used base R and *tidyverse* packages. Diel niche classification employed *Diel.Niche* (Gerber et al. 2024), and activity visualisation and overlap analyses used *overlap* and *activity* (Ridout and Linkie 2009; Meredith et al. 2024). Sunrise and sunset times were calculated with *suncalc* (Thieurmel and Elmarhraoui 2022).

Camera-trap data contain sensitive location information and are not publicly available. All analysis scripts and derived data summaries required to reproduce the results are available in a public repository (see Data availability).

## Results

### Diel niche classification

**Fig. 3:**
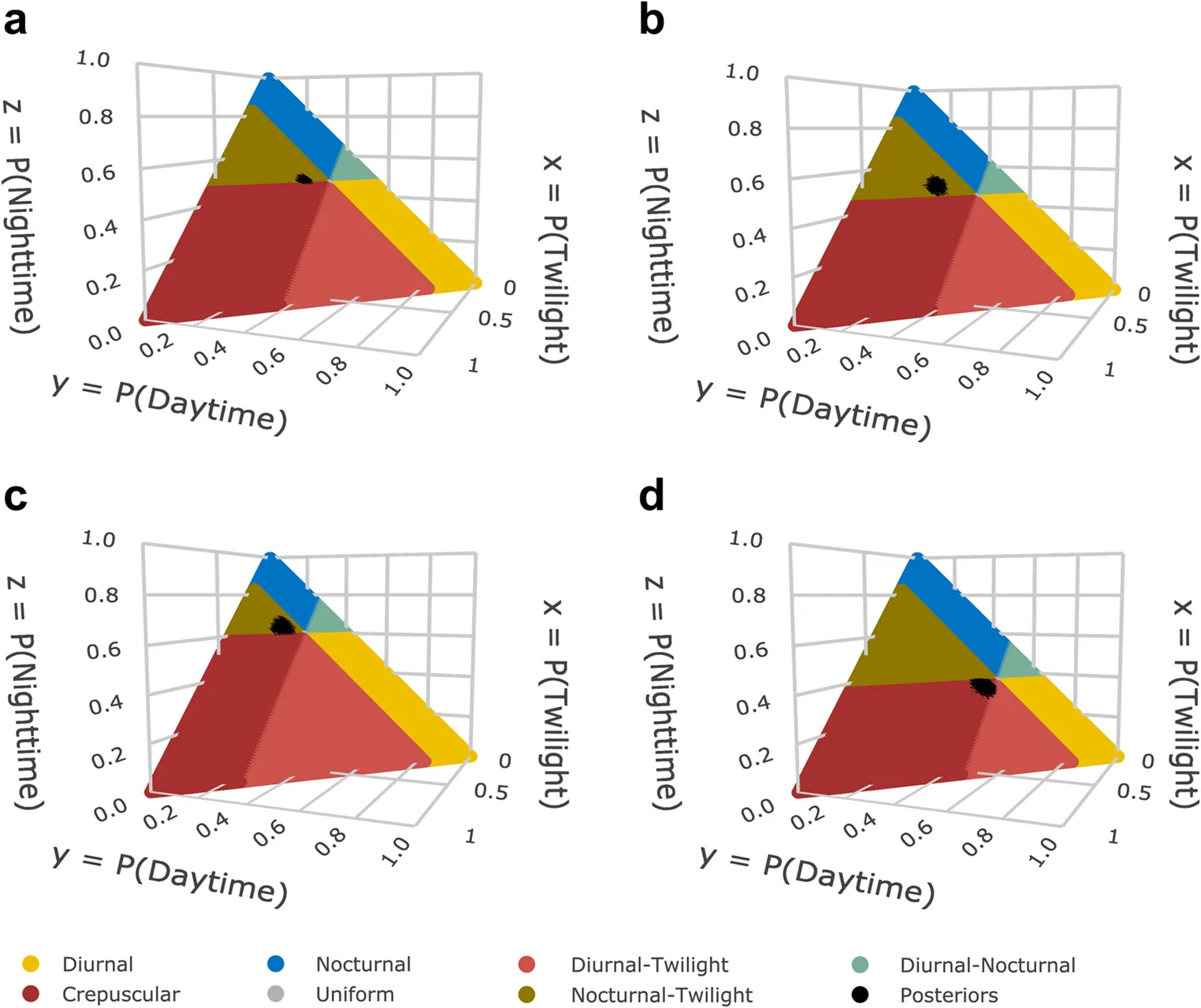
Diel niche classification of European fallow deer under the selection-hypothesis framework. Panels show results at the (**a**) population level and during the (**b**) rut (October), (**c**) post-rut (January), and (**d**) birthing (April) seasons. Triplots depict phase-use probabilities for day (*p*_*day*_), twilight (*p*_*twilight*_), and night (*p*_*night*_). Observed phase use is shown by black points (posteriors), plotted against diel phase availability (coloured regions), thereby accounting for seasonal variation in photoperiod (Gerber et al. 2024). Each black point represents one of 12,000 posterior draws

 Diel niche classification revealed a consistent bias toward low-light activity in European fallow deer. At the population level, activity was best described by a nocturnal–twilight niche, indicating preferential use of night and twilight relative to their availability (Fig. 3a, Table S6–S7). Season-specific analyses showed that this low-light bias persisted but varied in composition. During the rut and post-rut periods, activity was also best described as nocturnal–twilight, with strong posterior support (Fig. 3b, c, Table S6–S7). During the birthing period, activity shifted toward a predominantly twilight niche, while daytime remained underused relative to its availability (Fig. 3d, Table S6–S7).

Across seasons, uniform activity proportional to diel phase availability (i.e. equal use of phases) received no support (Table S7), indicating consistent behavioural selection for specific diel phases rather than activity patterns driven solely by photoperiod. Phase-use estimates showed disproportionate use of twilight and/or night and underuse of daytime relative to availability, indicating that seasonal differences were not attributable to photoperiod variation alone (Table S6).

### Seasonal and sex-specific variation in diel activity patterns

**Fig. 4:**
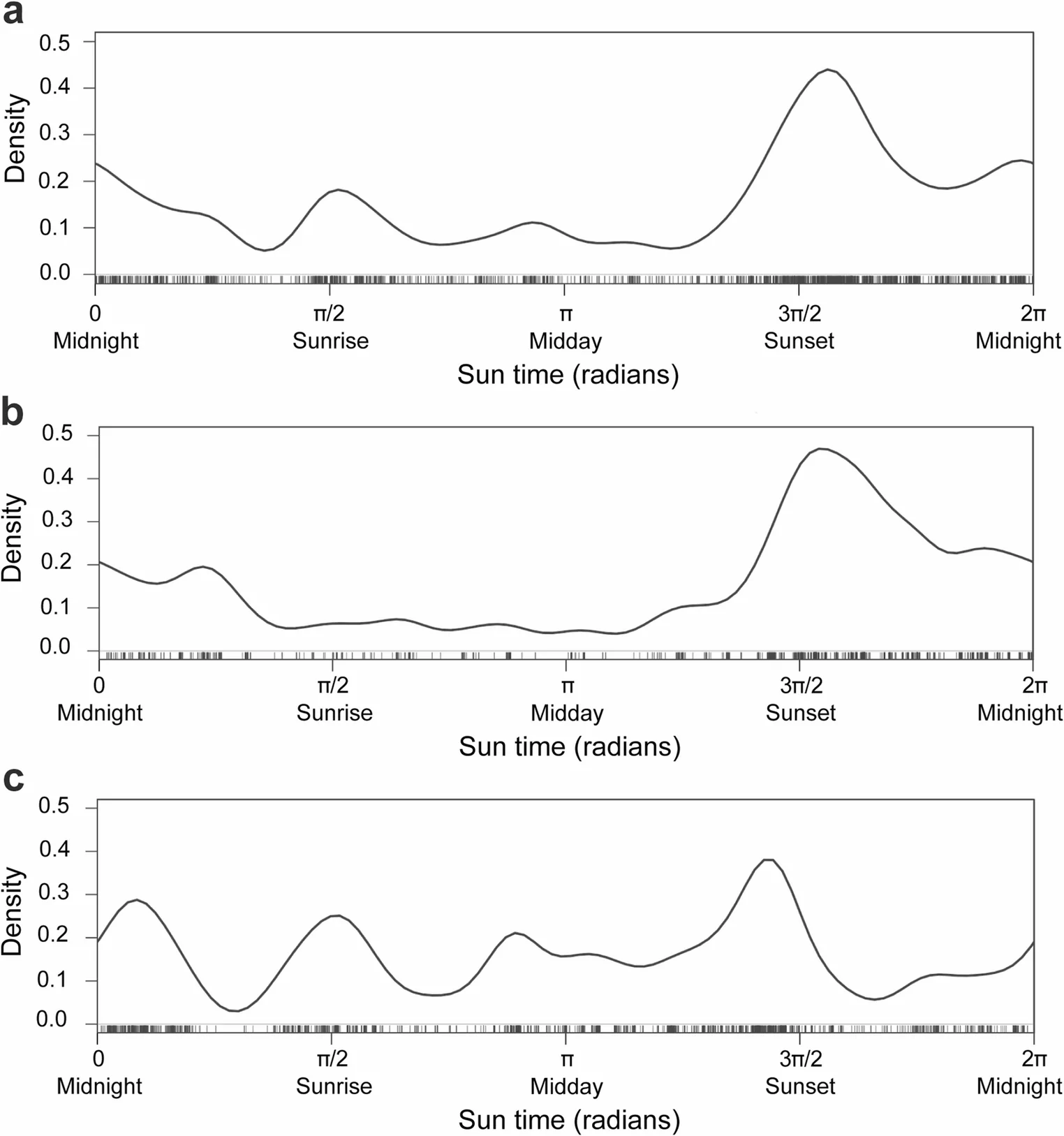
Seasonal variation in diel activity of European fallow deer. Panels show equinoctially anchored kernel density estimates for the (**a**) rut (October), (**b**) post-rut (January), and (**c**) birthing (April) seasons. Rug marks at the bottom of each panel indicate individual detections. Activity distributions are shown in relative sun time, with sunrise fixed at π/2, midday at π, sunset at 3π/2, and midnight at 0 and 2π, allowing direct comparison of activity timing across seasons over the 24-hour cycle

**Fig. 5:**
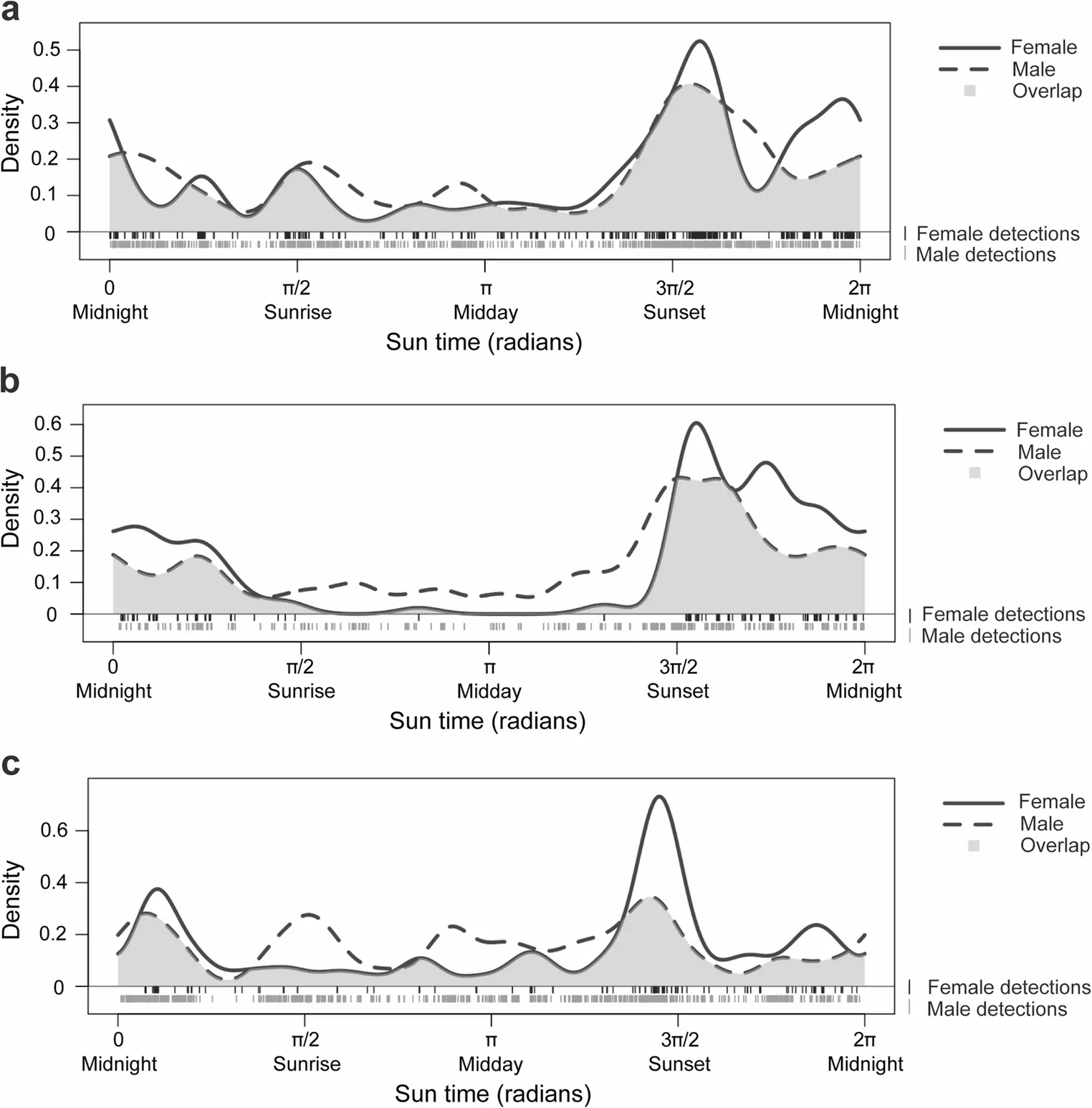
Sex-specific diel activity patterns of European fallow deer and temporal overlap within seasons. Panels show equinoctially anchored kernel density estimates for females (solid line) and males (dashed line) during the (**a**) rut (October), (**b**) post-rut (January), and (**c**) birthing (April) seasons. The shaded area represents the shared portion of the two activity distributions used to calculate the coefficient of overlap (Δ̂). The x-axis shows relative sun time in radians, with sunrise fixed at π/2, midday at π, sunset at 3π/2, and midnight at 0 and 2π. Rug marks indicate individual detections for each sex. Sex-specific activity kernels for males and females plotted separately are shown in Fig. S2

Equinoctially anchored kernel density estimates revealed seasonal changes in population-level activity patterns that were further resolved by sex-specific analyses (Figs. 4 and 5). During the rut, population-level activity was concentrated around evening twilight and night, with little daytime activity (Fig. 4a). Males and females showed broadly similar activity patterns, with closely aligned peaks around sunset and early night (Fig. 5a and Fig. S2). Accordingly, temporal overlap between sexes was highest during the rut (Δ̂ = 0.86, 95% CI: 0.80–0.88).

During the post-rut period, population-level activity remained predominantly nocturnal but was more broadly distributed across the night (Fig. 4b). Sex-specific analyses revealed greater differentiation than during the rut (Fig. 5b). Female activity was concentrated around sunset and night, with little activity around sunrise or during daytime, whereas males showed a broader temporal distribution extending further into daytime (Fig. S2). Temporal overlap consequently declined to Δ̂ = 0.71 (95% CI: 0.66–0.75).

During the birthing period, the population-level activity distribution showed increased use of twilight and daylight hours compared with the rut and post-rut periods (Fig. 4c). However, sex-specific analyses showed that this seasonal change was largely driven by males (Figs. 5c, S2). Females maintained a predominantly low-light activity pattern, with pronounced peaks around sunset and after midnight and limited daytime activity, whereas males showed a broader, multimodal distribution with increased twilight and daytime activity. Temporal overlap was lowest during the birthing period (Δ̂ = 0.69, 95% CI: 0.61–0.75).

Overall, the population-level seasonal shift toward greater twilight and daytime activity during the birthing period therefore masked contrasting sex-specific patterns: females remained predominantly active during low-light periods, whereas males showed greater temporal flexibility and increased daytime activity. Temporal overlap between the sexes declined from the rut through the post-rut to the birthing period, indicating increasing sexual segregation in diel activity outside the rut.

## Discussion

Our results demonstrate seasonal and sex-specific structure in the diel activity of fallow deer in a landscape that is characterized by daytime recreation and female-focused culling. Selection-based diel niche classification revealed a consistent population-level bias toward low-light activity, while kernel density analyses showed that seasonal changes in activity timing were consistent with redistribution within twilight and night, rather than a shift to diurnality. Although daytime activity increased during the birthing period, overall avoidance of daylight persisted. Importantly, sex-specific analyses revealed increasing temporal segregation outside the rut, with females maintaining persistent nocturnality and males exhibiting greater temporal flexibility, including increased daytime activity during the birthing period. These patterns occurred in the context of substantial but temporally variable recreational use and sex-biased management, but may also reflect intrinsic sex- and season-specific behavioural and energetic constraints. Below, we interpret these findings in the context of human disturbance, management, and reproductive constraints, and consider their implications for wildlife monitoring and population management.

### Selection for low-light activity in a human-dominated landscape

Selection-based diel niche classification revealed a consistent bias toward activity during twilight and night, indicating that fallow deer in the Amsterdamse Waterleidingduinen preferentially use low-light periods relative to their availability. This pattern supports our first prediction that fallow deer would exhibit an overall bias toward twilight and night activity in this human-dominated landscape. Importantly, this low-light bias persisted across seasons despite substantial variation in photoperiod, demonstrating that activity timing cannot be explained by changing day length alone. Instead, the results are consistent with behavioural selection against daytime activity and reduced temporal overlap with periods of recreational access (Gaynor et al. 2018). Such selection for low-light activity is widely reported in ungulates inhabiting human-dominated landscapes, where humans can function as a temporally structured source of risk, particularly where non-lethal human activity occurs alongside hunting or population management (Ciuti and Apollonio 2008; Coppes et al. 2017; Ikeda et al. 2019; Millien et al. 2024; Mori et al. 2025; Boer-Cueva et al. 2026). In the AWD, Waternet ticket-sales records indicate substantial seasonal variation in recreational use, with 16,475 day-admission tickets sold in April 2024, 30,075 in October 2024, and 13,198 in January 2025 (Waternet, unpublished data), corresponding to the birthing, rut, and post-rut sampling periods, respectively. These figures provide an index rather than complete visitor counts because not all visitors purchase a day-admission ticket. This seasonal variation in visitation did not show an obvious correspondence with the observed activity patterns, although the three sampling periods and coarse temporal resolution of the visitor data preclude direct assessment of this relationship. In contrast to this variation in intensity, the diel timing of recreational access is more predictable because public access is restricted to daylight hours. The observed selection for twilight and night is therefore consistent with reduced temporal overlap with recreational activity, as also reported for other deer species (Coppes et al. 2017; Millien et al. 2024), although our study was not designed to directly test the effect of recreation intensity on deer activity.

Within this overall crepuscular–nocturnal niche, twilight use was asymmetric, with activity concentrated more strongly around sunset than sunrise. In the AWD, evening twilight and the onset of night also coincide with the end of daily public access, potentially providing a period of reduced human disturbance that may reinforce this pattern (Millien et al. 2024; van der Spek 2024; Torretta et al. 2025). Variation in ambient light conditions caused by cloud cover or lunar illumination may influence activity patterns within twilight and night-time periods (Śmielak 2023), although these factors were not quantified in the present study. The persistence of a nocturnal–twilight niche across seasons has been reported in other fallow deer populations (Barton 2023) and suggests that selection against daytime activity persists even when energetic demands increase, such as during the rut or birthing period. In systems with stronger resource limitation, higher ungulate densities, greater competition, or predation risk, energetic demands may impose different constraints on activity scheduling than those observed in the AWD. By explicitly accounting for diel phase availability, the selection-hypothesis framework (Gerber et al. 2024) strengthens inference by demonstrating that low-light activity reflects active behavioural selection rather than a by-product of seasonal photoperiod change. Together, these findings highlight how human disturbance, management, and abiotic constraints jointly shape low-light activity in protected but intensively used landscapes.

### Seasonal and sex-specific variation in diel activity

Population-level activity patterns varied seasonally, but the sex-specific analyses showed that these changes reflected contrasting temporal responses of males and females. During the rut, both sexes exhibited similar low-light activity patterns and high temporal overlap, consistent with the reduction in sexual segregation expected during the mating period (Ciuti et al. 2006; Barton 2023). After the rut, temporal overlap declined as females maintained a predominantly nocturnal activity pattern whereas males showed a broader temporal distribution. This divergence was most apparent during the birthing period, when the population-level increase in twilight and daytime activity was largely driven by males, while females remained predominantly active during low-light periods. Thus, interpreting seasonal variation from pooled population-level activity alone would obscure an important component of the observed behavioural variation: males and females differed in how their activity changed across seasons. The increasing temporal segregation outside the rut complements well-documented spatial and habitat segregation between the sexes in fallow deer and other sexually dimorphic ungulates (Bowyer and Kie 2004; Imperio et al. 2020; Cueva-Hurtado et al. 2024; Comte et al. 2025).

Several non-mutually exclusive mechanisms could contribute to these sex-specific seasonal patterns. Following the energetically demanding rut, males may broaden their activity as feeding resumes after the marked reduction in food intake during the rut, potentially contributing to the recovery of depleted energy reserves (Jopson et al. 1997; Newman et al. 1998). This recovery may continue into spring, when increasing forage availability could further favour greater temporal flexibility. Females, in contrast, face the energetic requirements of gestation and subsequent lactation and may differ from males in their allocation of time and activity (Barton 2023). Seasonal differences in reproductive and energetic constraints may therefore contribute to the divergence in male and female activity patterns, alongside sex-specific responses to human disturbance.

Human activities may additionally contribute to these patterns, but their effects cannot be isolated with our study design. Recreational access occurs throughout the year and is restricted to daylight hours, although its intensity varies substantially among seasons and days. Population control provides an additional, seasonally restricted source of disturbance and mortality risk: culling occurs from November to March and disproportionately targets females and fawns. Female-focused culling could therefore contribute to greater avoidance of periods associated with human activity, while management activities may also affect males through disturbance and perceived mortality risk (Grignolio et al. 2011; Delisle et al. 2024). Greater sensitivity to human disturbance has also been reported for female fallow deer, particularly during the fawning and early-lactation period (Ciuti et al. 2004; Ciuti and Apollonio 2008), and could contribute to the sex-specific patterns observed here, although this mechanism cannot be evaluated directly with our data. Comparable sex-specific responses to human disturbance and hunting have been reported in other ungulate systems (Ikeda et al. 2019; Chitwood et al. 2022).

These mechanisms cannot be disentangled in the present study. In particular, we lacked a control population without culling or a before–after control–impact design, and therefore cannot attribute the greater nocturnality of females or its seasonal variation specifically to female-focused population control. Moreover, recreation, culling, reproductive state, energetic requirements, and sex-specific risk sensitivity may interact rather than operate independently. The observed patterns should therefore be interpreted as sex-specific seasonal variation occurring within a landscape characterised by temporally variable daytime recreation and sex-biased population management, rather than as evidence for a causal effect of either form of human disturbance. Nevertheless, the decline in temporal overlap outside the rut demonstrates that sexual segregation in fallow deer extends to the diel dimension and that population-level activity patterns can mask ecologically relevant differences between the sexes.

### Implications for wildlife monitoring and management

The seasonal and sex-specific structure in diel activity documented in this study has direct implications for wildlife monitoring and management in human-dominated landscapes. Our results show that population-level summaries of activity can mask meaningful temporal differences between sexes, particularly outside the rut. Monitoring approaches based on direct observations or other methods that are sensitive to animal activity may mask sex-specific differences in activity and detectability when observations are pooled across sexes, potentially underrepresenting more nocturnal individuals such as females. Consequently, assessments of habitat use, grazing pressure, and wildlife impacts may be biased when temporal variation in activity is not considered. Similar concerns regarding monitoring bias have been raised in other ungulate studies, where nocturnality reduces detectability during standard survey periods (Coppes et al. 2017; Chitwood et al. 2022). Continuous camera-trap monitoring offers a valuable opportunity to quantify activity patterns throughout the full 24-hour cycle and to identify behavioural differences that remain hidden in conventional surveys.

These considerations are particularly relevant for monitoring programmes that rely on visual counts during specific periods of the day. In the Amsterdamse Waterleidingduinen, for example, annual population estimates are based on standardized dawn and dusk driving transects conducted during the birthing season. Our results suggest that both temporal segregation in activity and spatial segregation in habitat use between males and females could influence detectability and therefore affect estimates of population structure and abundance if not adequately considered in survey design.

For management, our findings indicate that sex- and season-specific activity patterns should be considered when determining the timing and methods of population-control activities. In systems such as the AWD, where population control primarily targets females and fawns, differences in diel activity between sexes may affect when target animals are most readily encountered and therefore the effort required for management, without necessarily determining its effectiveness. Incorporating information on diel activity into management planning could therefore help align control strategies with the behaviour of target groups and support more evidence-based management of large herbivores.

### Methodological considerations and limitations

This study relied on camera-trap detections to infer diel activity patterns, which capture the timing of activity but do not provide information on individual identity or movement paths. As a result, repeated detections may reflect activity of the same individuals, although sensitivity analyses indicated that time-to-independence filtering had limited influence on qualitative patterns of activity. Activity classifications were based on temporal occurrence rather than direct behavioural observations, and resting or inactive states may not always be distinguishable from low movement rates. Nevertheless, camera trapping remains a widely applied and effective approach for assessing diel activity in free-ranging mammals (Wearn and Glover-Kapfer 2017; Devarajan et al. 2025).

Our inference is also specific to a system characterised by substantial but temporally variable recreational use, absence of large predators, and long-standing sex-biased management. Although recreational access is restricted to daylight hours, its intensity varies seasonally and among days. Monthly ticket-sales data provide an index of seasonal variation in visitation, but we did not have visitor counts at the temporal and spatial resolution required to quantify the exposure of individual deer or camera locations to recreational activity. Consequently, we could not directly test how variation in recreation intensity influenced deer activity. Similarly, the absence of a control population without culling or a before–after control–impact design prevented us from separating potential effects of female-focused culling from intrinsic seasonal differences in male and female behaviour and physiology. While these conditions provide a useful context for examining behavioural responses to human disturbance, extrapolation to systems with different predator communities or management regimes should be made cautiously. Finally, although the selection-based diel niche framework accounts for seasonal variation in light availability, it assumes that detections are proportional to activity within diel phases, which may be influenced by habitat structure or camera placement. These considerations do not undermine the main conclusions but highlight avenues for future work combining temporal analyses with spatial and individual-based data.

## Conclusions

This study demonstrates that diel activity of European fallow deer in a human-dominated landscape is strongly structured by both season and sex. Using a selection-based diel niche framework combined with continuous activity analyses, we show that fallow deer consistently select low-light periods, while seasonal changes in activity are expressed as redistribution within twilight and night, and to a lesser extent daytime, rather than shifts to diurnality. Importantly, sex-specific analyses revealed increasing temporal segregation outside the rut, with females maintaining persistent nocturnality and males exhibiting greater flexibility in activity timing, particularly during the birthing period. These contrasting patterns would have been obscured by population-level analyses alone, highlighting the importance of resolving diel activity by sex. The observed patterns occurred within a landscape characterised by temporally variable daytime recreation and sex-biased population management, but their underlying mechanisms cannot be disentangled from intrinsic sex- and season-specific behavioural and energetic constraints. Our findings underscore the importance of incorporating season- and sex-specific activity patterns into wildlife monitoring and management practices in human-dominated landscapes.

## Supplementary Information

Below is the link to the electronic supplementary material.

Supplementary Material 1 (DOCX 452 KB)

## Data Availability

All processed data and R scripts supporting the findings of this study are publicly available on Zenodo (https://doi.org/10.5281/zenodo.18694720). Raw camera-trap images are not shared due to data sensitivity and site protection considerations.
