# Supplementary material for: Low-light activity in fallow deer shows sex-specific seasonal changes in a managed landscape
Source: Mamm Res. 2026 Sep 29;71(4):82. doi: 10.1007/s13364-026-00903-8 (PMC13624009; doi:10.1007/s13364-026-00903-8)
Supplement: Supplementary file 1 [file 13364_2026_903_MOESM1_ESM.docx]

**SUPPLEMENTARY INFORMATION**

**Low-light activity in fallow deer shows sex-specific seasonal changes in a managed landscape**

**Akke de Lange^1^, Luc H W T Geelen^2^ & W Daniel Kissling^1,*^**

^1^ Institute for Biodiversity and Ecosystem Dynamics (IBED), University of Amsterdam, P.O. Box 94240, 1090 GE Amsterdam, The Netherlands

^2^ Waternet, Vogelenzangseweg 21, 2114, BA, Vogelenzang, the Netherlands

*****	Correspondence: [W.D.Kissling@uva.nl](mailto:W.D.Kissling@uva.nl); Tel.: +31 (0)20 525 6635

**Text S1: Sensitivity analysis of time-to-independence filtering**

Camera-trap studies commonly apply a time-to-independence filter (TTIF) to reduce repeated detections of the same individuals within short time intervals (Yasuda, 2004; Peral et al., 2022). However, TTIFs were originally developed for abundance or occupancy analyses and may bias estimates of diel activity by discarding biologically meaningful detections and flattening activity distributions (Peral et al., 2022). To assess whether TTIF application influenced inference in this study, we conducted a sensitivity analysis comparing results obtained from the unfiltered dataset with those from a dataset filtered using a fixed time-to-independence threshold.

Inter-detection intervals were examined by plotting the cumulative number of detections per camera location against increasing time thresholds following Yasuda (2004). Across seasons and sexes, a threshold of 15 minutes corresponded to the inflection point of the cumulative detection curve (Fig. A1) and fell within the range commonly applied in camera-trap studies of social ungulates. Based on this assessment, detections occurring within 15 minutes at the same camera location were merged into a single event.

**Fig. S1** Total number of fallow deer detections per camera location plotted against increasing inter-detection interval thresholds. In both the birthing and post-rut seasons, the curve shows a clear inflection at 15 minutes, indicating diminishing gains in reducing repeated detections beyond this interval. Although the rut curve does not fully asymptote, 15 minutes represents the first threshold at which the rate of change between successive intervals falls below 5%, following the criterion proposed by Yasuda (2004)

Applying the 15-minute TTIF reduced the total number of detections by approximately 60%, with particularly strong reductions for females during the birthing season (Table A1). Seasonal activity overlap estimates derived from equinoctially anchored kernel density curves were broadly similar in magnitude between filtered and unfiltered datasets, but confidence intervals were consistently wider after filtering (Table A2), reflecting reduced sample size and dampened activity peaks. In addition, TTIF application reduced posterior support for the most supported diel niche hypothesis under the selection framework across all seasons but only altered the best-supported hypothesis in the birthing period (Table A3).

Because TTIF application consistently reduced sample size, dampened activity structure, and weakened inferential precision, all main analyses were conducted using the unfiltered (full) dataset. This approach is consistent with recent recommendations that time-to-independence filtering can bias camera-trap–based activity analyses and should be avoided when quantifying diel activity patterns (Peral et al., 2022).

**Table S1**	Number of fallow deer detections before and after applying a 15-minute time-to-independence filter (TTIF), summarised by season and sex

| Season | All | Females only | Males only |
| --- | --- | --- | --- |
| *Before applying TTIF (full dataset)* | | | |
| Rut | 1822 | 562 | 1260 |
| Post-rut | 913 | 235 | 678 |
| Birthing | 1379 | 152 | 1227 |
| Total | 4114 | 949 | 3165 |
|  |  |  |  |
| *After applying a 15 min TTIF* | | | |
| Rut | 889 | 219 | 670 |
| Post-rut | 586 | 85 | 501 |
| Birthing | 271 | 82 | 189 |
| Total | 1746 | 386 | 1360 |

**Table S2**	Seasonal overlap coefficients (Δ̂) with 95% confidence intervals (CI) before and after applying a 15-minute time-to-independence filter (TTIF)

| Season pair | Δ̂ before applying TTIF (95% CI) | CI width | Δ̂ after applying a 15 min TTIF (95% CI) | CI width |
| --- | --- | --- | --- | --- |
| Post rut vs. birthing | 0.571 (0.532–0.600) | 0.068 | 0.606 (0.537–0.656) | 0.120 |
| Post rut vs. rut | 0.857 (0.819–0.872) | 0.053 | 0.863 (0.786–0.878) | 0.091 |
| Birthing vs. rut | 0.664 (0.632–0.687) | 0.055 | 0.720 (0.668–0.757) | 0.089 |

**Table S3**	Most supported diel niche hypothesis and posterior probability under the selection framework, before and after applying a 15-minute time-to-independence filter (TTIF)

| Season | Diel selection before applying TTIF | Posterior | Diel selection after applying a 15 min TTIF | Posterior | Change in posterior support |
| --- | --- | --- | --- | --- | --- |
| Rut | Night-twilight | 1.0000 | Night-twilight | 0.9304 | −0.0696 |
| Post rut | Night-twilight | 0.9666 | Night-twilight | 0.5850 | −0.3817 |
| Birthing | Twilight | 0.9832 | Day-twilight | 0.9333 | −0.0499 |

**Text S2: Diel niche hypotheses, constraint definitions, and prior specifications**

**Overview of the diel niche framework**

Diel activity was classified using a hypothesis-based diel niche framework that evaluates animal activity in relation to the availability of diel phases (day, twilight, night), rather than absolute clock time (Gerber et al., 2024). This framework explicitly accounts for seasonal variation in photoperiod and allows discrimination between apparent activity shifts driven by changing light availability and behavioural selection for specific diel phases (Nouvellet et al., 2012; Gerber et al., 2024). The approach, termed 'selection hypothesis' (sensu Gerber et al., 2024), is particularly suited to seasonal comparisons and temperate or high-latitude systems where day length varies substantially across the year.

For each grouping (population level, season, and sex), observed activity was summarised as the proportion of detections occurring in each diel phase. These proportions were evaluated against phase-specific availability (Table B1) to test competing hypotheses describing diel niche use.

**Table S4** Seasonal availability of diel phases in the study area. For each sampled season representing the rut, post-rut, and birthing period, the table reports the total number of hours classified as twilight, day, and night, together with their percentage of the 24-hour cycle. Twilight duration was held constant across seasons (4 h; 16.7%), whereas day length increased from post-rut to birthing (5.9 to 11.3 h), with a corresponding decrease in night length (14.1 to 8.7 h)

| Month | Twilight hours | Twilight percentage | Day  hours | Day percentage | Night  hours | Night percentage |
| --- | --- | --- | --- | --- | --- | --- |
| Rut | 4 | 16.7 % | 9.4 | 38.9 % | 10.6 | 44.4 % |
| Post-rut | 4 | 16.7 % | 5.9 | 24.4 % | 14.1 | 58.9 % |
| Birthing | 4 | 16.7 % | 11.3 | 46.9 % | 8.7 | 36.4 % |

**Observed phase use and availability**

Let *y* = (*y_D_*, *y_T_*, *y_N_*) denote the observed counts of detections occurring during day (D), twilight (T), and night (N), respectively, for a given grouping. Let *n* = *y_D_* + *y_T_* + *y_N_* be the total number of detections.

Let *a* = *a_D_*, *a_T_*, *a_N_* denote the proportional availability of each diel phase, defined as the fraction of the 24-hour cycle occupied by each phase during the relevant season. By definition,

*a_D_* + *a_T_* + *a_N_* = 1

Observed phase use is modelled as a multinomial process:

*y* ~ Multinomial(*n*, *p*)

where *p* = (*p_D_*, *p_T_*, *p_N_*) represents the true underlying probability of activity in each phase.

**Diel niche hypotheses**

Candidate diel niche hypotheses were formulated as biologically interpretable constraints on the phase-use probabilities *p*, following Gerber et al. (2024). The following seven hypotheses were evaluated, i.e. implementing the 'selection hypothesis' framework from Gerber et al. (2024):

1. **Uniform**
   Activity proportional to phase availability (i.e. equal use):

*p_D_* = *a_D_*, *p_T_* = *a_T_*, *p_N_* = *a_N_*

2. **Diurnal**
   Preferential use of day:

*p_D_* > *p_T_*, *p_D_* > *p_N_*

3. **Nocturnal**
   Preferential use of night:

*p_N_* > *p_T_*, *p_N_* > *p_D_*

4. **Crepuscular (twilight)**
   Preferential use of twilight:

*p_T_* > *p_D_*, *p_T_* > *p_N_*

5. **Nocturnal– twilight**
   Combined preference for night and twilight over day:

*p_N_* = *p_T_* > *p_D_*

6. **Diurnal– twilight**
   Combined preference for day and twilight over night:

*p_D_* = *p_T_* > *p_N_*

7. **Diurnal–nocturnal**

Avoidance of twilight, with activity concentrated during both day and night:

*p_D_* = *p_N_* > *p_T_*

These hypotheses span a continuum from strict single-phase specialisation to mixed or graded activity strategies, allowing flexible classification of diel activity patterns.

**Constraint implementation**

Hypotheses were implemented as inequality or equality constraints on *p* within a Bayesian multinomial framework via the ‘Diel.Niche’ R package (Gerber et al., 2024). For each hypothesis, the parameter space of *p* was restricted to values satisfying the corresponding constraints. Equality constraints (e.g. *p_N_* = *p_T_*) were enforced by reparameterization, whereas inequality constraints (e.g. *p_D_* ≥ *p_T_*, *p_D_* ≥ *p_N_*) were enforced by truncation of the posterior distribution.

All hypotheses respected the simplex constraint:

*p_D_* + *p_T_* + *p_N_* = 1, *p_i_* ≥ 0

Formal constraint definitions and parameter bounds for each diel niche hypothesis are provided in Table B2. These constraints represent a mathematical implementation of the biological hypotheses described by Gerber et al. (2024) as implemented within the Bayesian constrained-multinomial framework of the ‘Diel.Niche’ R package, using linear equality and inequality formulations that are standard in constrained statistical inference (Silvapulle and Sen, 2004).

**Table S5** Diel niche hypotheses evaluated in this study and their corresponding constraint definitions. Hypotheses are expressed as equality and inequality relationships among the probabilities of activity during day (*p_D_*), twilight (*p_T_*), and night (*p_N_*). All hypotheses were evaluated under the simplex constraint *p_D_* + *p_T_* + *p_N_* = 1. *I_3_* is the 3 × 3 identity matrix. Implementation is available in the ‘Diel.Niche’ R package (Gerber et al., 2024)

| Hypothesis | Equality constraints *E***p** = **d** | Inequality constraints *A***p** ≥ **0** |
| --- | --- | --- |
| Uniform | **p** = **a** (i.e., $E=I_{3},\text{ }\mathbf{d}=\mathbf{a}$) | — |
|  |  |  |
| Diurnal | — | $\left[ \begin{matrix} 1 & -1 & 0 \\ 1 & 0 & -1 \end{matrix} \right]$ **p** ≥ **0** (i.e. *p_D_* ≥ *p_T_*, *p_D_* ≥*p_N_*) |
|  |  |  |
| Nocturnal | — | $\left[ \begin{matrix} -1 & 0 & 1 \\ 0 & -1 & 1 \end{matrix} \right]$ **p** ≥ **0** (i.e. *p_N_* ≥ *p_D_*, *p_N_* ≥*p_T_*) |
|  |  |  |
| Crepuscular (twilight) | — | $\left[ \begin{matrix} -1 & 1 & 0 \\ 0 & 1 & -1 \end{matrix} \right]$ **p** ≥ **0** (i.e. *p_T_* ≥ *p_D_*, *p_T_* ≥*p_N_*) |
|  |  |  |
| Nocturnal–twilight | $\left[ \begin{matrix} 0 & 1 & -1 \end{matrix} \right]$**p** = 0 (i.e., *p_T_* = *p_N_*) | $\left[ \begin{matrix} -1 & 1 & 0 \end{matrix} \right]$**p** ≥ 0 (i.e., *p_T_* ≥ *p_D_*; with equality above implies *p_N_* = *p_T_* ≥ *p_D_* |
|  |  |  |
| Diurnal–twilight | $\left[ \begin{matrix} 1 & -1 & 0 \end{matrix} \right]$ **p** = 0 (i.e., *p_D_* = *p_T_*) | $\left[ \begin{matrix} 1 & 0 & 1 \end{matrix} \right]$**p** ≥ 0 (i.e., *p_D_* ≥ *p_N_*; with equality above implies *p_D_* = *p_T_* ≥ *p_N_* |
|  |  |  |
| Diurnal–nocturnal | $\left[ \begin{matrix} 1 & 0 & -1 \end{matrix} \right]$ **p** = 0 (i.e., *p_D_* = *p_N_*) | $\left[ \begin{matrix} 0 & -1 & 1 \end{matrix} \right]$**p** ≥ 0 (i.e., *p_N_* ≥ *p_T_*; with equality above implies *p_D_* = *p_N_* ≥ *p_T_* |

**Prior specifications**

A symmetric Dirichlet prior was specified for *p*:

*p* ~ Dirichlet(1,1,1)

This prior assigns equal probability to all possible activity distributions and is non-informative with respect to diel phase preference. For constrained hypotheses, this prior was truncated to the admissible region defined by the hypothesis-specific constraints (Table B2). No informative priors were placed on activity preferences to avoid biasing classification toward expected diel strategies. These specifications follow the implementation in the ‘Diel.Niche’ R package (Gerber et al., 2024).

**Posterior inference and hypothesis selection**

Posterior distributions of *p* were estimated using the Markov chain Monte Carlo (MCMC) sampling from the ‘Diel.Niche’ R package (Gerber et al., 2024). For each hypothesis, posterior support was quantified as the posterior probability that the data arose from the constrained parameter space of that hypothesis, conditional on the observed detections and phase availability.

Hypotheses were compared within a Bayesian selection framework, and the hypothesis with the highest posterior probability was retained as the best-supported diel niche. Posterior probabilities were interpreted as relative support among competing hypotheses rather than absolute measures of model adequacy.

**Model diagnostics and robustness**

Convergence of MCMC chains was assessed visually and using standard diagnostics (trace plots and effective sample sizes). Sensitivity to data filtering was evaluated separately via time-to-independence filtering (see Appendix A). Across seasons and sexes, diel niche classification was generally robust to filtering, although posterior support weakened under reduced sample sizes.

**Quantitative support for diel niche classification**

**Table S6** Observed phase-use probabilities *p* (mean ± 95% credible interval) and proportional diel phase availability *a* for European fallow deer at the population level and by season, estimated under the selection hypothesis framework (sensu Gerber et al., 2024)

| Grouping | Phase | Phase use  (*p*) | 95% credible interval | Phase availability (*a*) |
| --- | --- | --- | --- | --- |
| Population | Day | 0.278 | 0.265–0.290 | 0.368 |
|  | Twilight | 0.250 | 0.238 0.262 | 0.167 |
|  | Night | 0.472 | 0.466–0.484 | 0.465 |
| Rut | Day | 0.230 | 0.211–0.250 | 0.389 |
|  | Twilight | 0.274 | 0.254–0.295 | 0.167 |
|  | Night | 0.495 | 0.473–0.518 | 0.444 |
| Post-rut | Day | 0.159 | 0.137–0.181 | 0.244 |
|  | Twilight | 0.230 | 0.205–0.254 | 0.167 |
|  | Night | 0.611 | 0.590–0.639 | 0.589 |
| Birthing | Day | 0.427 | 0.403–0.454 | 0.469 |
|  | Twilight | 0.240 | 0.217–0.262 | 0.167 |
|  | Night | 0.333 | 0.309–0.357 | 0.364 |

**Table S7** Posterior probabilities for competing diel niche hypotheses under the selection hypothesis framework, estimated for European fallow deer at the population level and by season. The most supported hypothesis for each grouping is highlighted in bold

| Grouping | Uniform | Diurnal | Crepuscular | Nocturnal | Nocturnal–Twilight | Diurnal–Twilight | Diurnal–Nocturnal |
| --- | --- | --- | --- | --- | --- | --- | --- |
| Population | 0 | 0 | 0.26715530 | 0 | **0.73284470** | 0 | 0 |
| Rut | 0 | 0 | 0 | 0 | **1** | 0 | 0 |
| Post-rut | 0 | 0 | 0.03243722 | 0 | **0.96756278** | 0 | 0 |
| Birthing | 0 | 0 | **0.98692342** | 0 | 0.01097074 | 0.00210584 | 0 |

**Sex-specific activity kernels**

**Fig. S2** Sex-specific diel activity patterns of European fallow deer across seasons. Panels show kernel density estimates for males (left) and females (right) during the rut, post-rut, and birthing periods, based on equinoctially anchored relative sun time, with sunrise fixed at π/2, midday at π, sunset at 3π/2, and midnight at 0 and 2π, allowing direct comparison of activity timing across seasons

**References**

Gerber, B. D., Devarajan, K., Farris, Z. J., & Fidino, M. (2024). A model-based hypothesis framework to define and estimate the diel niche via the ‘Diel.Niche’ R package. *Journal of Animal Ecology*, 93(2), 132-146. <https://doi.org/10.1111/1365-2656.14035>.

Nouvellet, P., Rasmussen, G. S. A., Macdonald, D. W., & Courchamp, F. (2012). Noisy clocks and silent sunrises: measurement methods of daily activity pattern. *Journal of Zoology*, 286(3), 179-184. <https://doi.org/10.1111/j.1469-7998.2011.00864.x>.

Peral, C., Landman, M., & Kerley, G. I. H. (2022). The inappropriate use of time-to-independence biases estimates of activity patterns of free-ranging mammals derived from camera traps. *Ecology and Evolution*, 12(10), e9408. <https://doi.org/10.1002/ece3.9408>.

Silvapulle, M. J., & Sen, P. K. (2004). Constrained statistical inference: Order, inequality, and shape constraints. New Jersey, USA: John Wiley & Sons.

Yasuda, M. (2004). Monitoring diversity and abundance of mammals with camera traps: a case study on Mount Tsukuba, central Japan. *Mammal Study*, 29(1), 37-46. <https://doi.org/10.3106/mammalstudy.29.37>.
